# Supplementary material for: Cold Atmospheric Plasma Restores Paclitaxel Sensitivity to Paclitaxel-Resistant Breast Cancer Cells by Reversing Expression of Resistance-Related Genes
Source: Cancers (Basel). 2019 Dec 13;11(12):2011. doi: 10.3390/cancers11122011 (PMC6966695; doi:10.3390/cancers11122011)
Supplement: Supplementary file 1 [file cancers-11-02011-s001.pdf]

cancers

Supplementary Materials

Table S1. Twenty genes that were deregulated in the MCF7/TxR but recovered by CAP.

Gene Symbol	Accession No.	Description	Fold change	
			MCF-7 vs. MCF-7/TxR	MCF-7/TxR vs. MCF-7/TxR + CAP
TMEM164	NM_017698.2	transmembrane protein 165	−2.471006	1.304404
RPS6KA2	NM_021135.6	ribosomal protein S6 kinase A2	−2.213120	1.529600
SLC44A1	NM_080546.5	solute carrier family 44 member 1	−1.495163	1.900823
TLE4	NM_001282748.1	TLE family member 4, transcriptional corepressor	−2.565393	2.905084
DAGLA	NM_006133.3	diacylglycerol lipase alpha	−1.810102	1.780053
ATF6B	NM_004381.5	activating transcription factor 6 beta	−1.729802	2.097785
FERMT3	NM_031471.6	fermitin family member 3	−1.748523	2.241378
KCMF1	NM_020122.5	potassium channel modulatory factor 1	−1.335370	1.607224
PHKA1	NM_002637.4	phosphorylase kinase regulatory subunit alpha 1	−1.922488	1.876008
LRRIQ3	NM_001105659.2	leucine rich repeats and IQ motif containing 3	−1.371887	1.346779
ESYT3	NM_001322831.2	extended synaptotagmin 3	−1.085915	−1.582270
UBXN8	NM_005671.4	UBX domain protein 8	1.563599	−1.525176
CNTD2	NM_024877.4	cyclin N-terminal domain containing 2	2.149036	−1.797696
ZNF304	NM_001290318.1	zinc finger protein 304	2.056123	−1.683319
GOLM1	NM_016548.4	golgi membrane protein 1	2.085563	−3.506264
CEACAM1	NM_001712.5	CEA cell adhesion molecule 1	4.416401	−1.551493
WNT7B	NM_058238.3	Wnt family member 7B	2.310505	−1.522427
KIF13B	NM_015254.4	kinesin family member 13B	3.209376	−1.973119
AQP3	NM_004925.5	aquaporin 3 (Gill blood group)	1.822655	−1.575980
IFIT1	NM_001548.5	interferon induced protein with tetratricopeptide repeats 1	8.551776	−1.313550

Table S2. Information of primers employed in this study.

Gene		Sequence (5′–3′)	Supplier
KIF13B	F :	AGCTACAAGGATATTGAGTCGTT	Bionics
	R :	TCTCTCCAGATGTCCCAGAC	
CEACAM1	F :	CCATCATGCTGAACGTAAACTAT	
	R :	CTTGCCCTGCCGGTCTTC	
GOLM1	F :	AGTTCTCCTACGACCTGAGC	
	R :	TTGGAGCTGCTGTCTCTGG	
TLE4	F :	TTCCTGTCCCAAGAGCACC	
	R :	GCCTGGAGTTGTTGCCCAA	
PHKA1	F :	ATGATCAGACAGGTGGATAAAGT	
	R :	TATGGAGTCCTGAGGCAGTC	
DAGLA	F :	CCCAGTCAGATGCCTACTCA	
	R :	GTCATTGTTTGCCTCGTCCA	

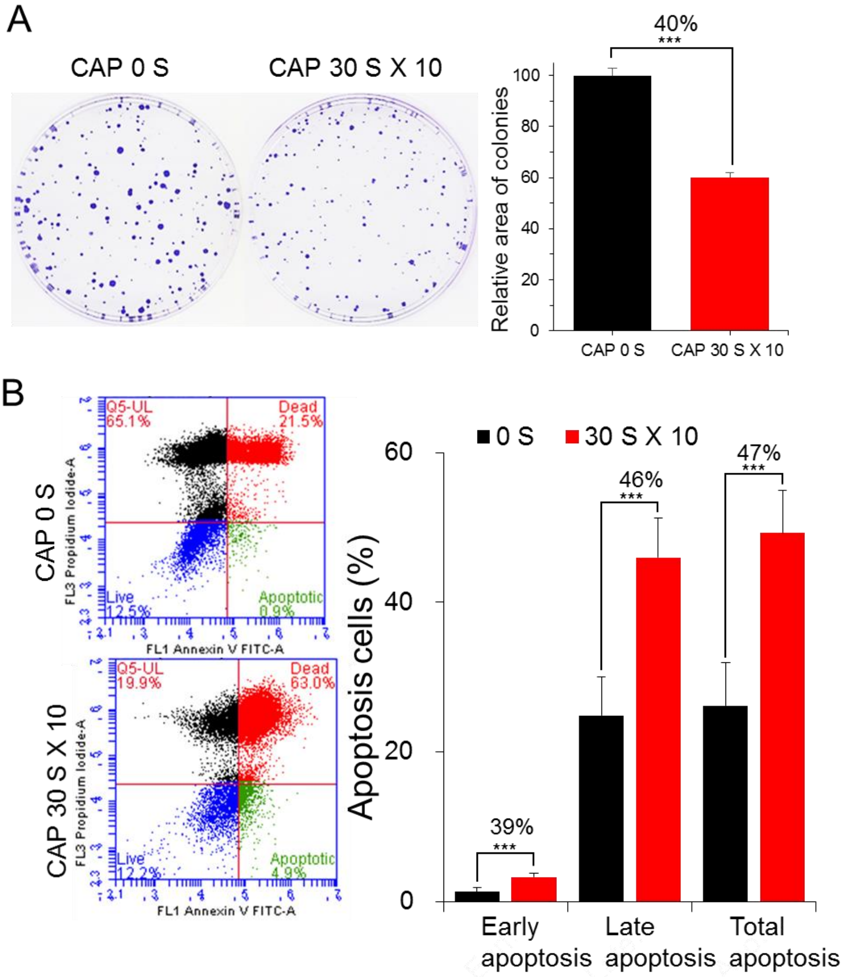

Figure S1. Inhibition of cell growth for MCF-7 by CAP. MCF-7 cells were treated with plasma for 10 times (30 sec each time with an hour interval between exposures), and cell growth and apoptosis were analyzed by colony formation assay (**A**) and FACS (**B**), respectively. All experiments were performed in triplicate and indicated as mean ± SE. *** $p < 0.001$.

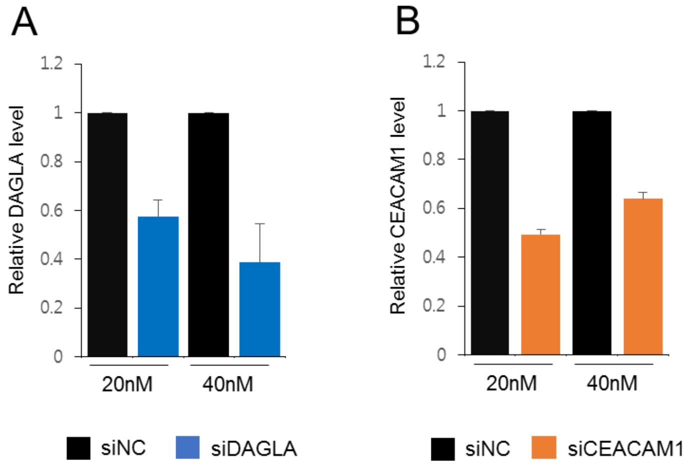

Figure S2. Induction of downregulation of DAGLA and CEACAM1 in MCF-7/TxR cells. Cultured cells were transiently transfected with a gene-specific siRNA to downregulate DAGLA (**A**) and CEACAM1 (**B**) in MCF-7/TxR cells. Expression of the gene was determined by real-time RT-PCR. Based on the downregulated level, 40 and 20 nM of siRNA were selected for DAGLA and CEACAM1, respectively. All experiments were performed in triplicate and indicated as mean ± SE. NC, negative control vector, or siRNA.

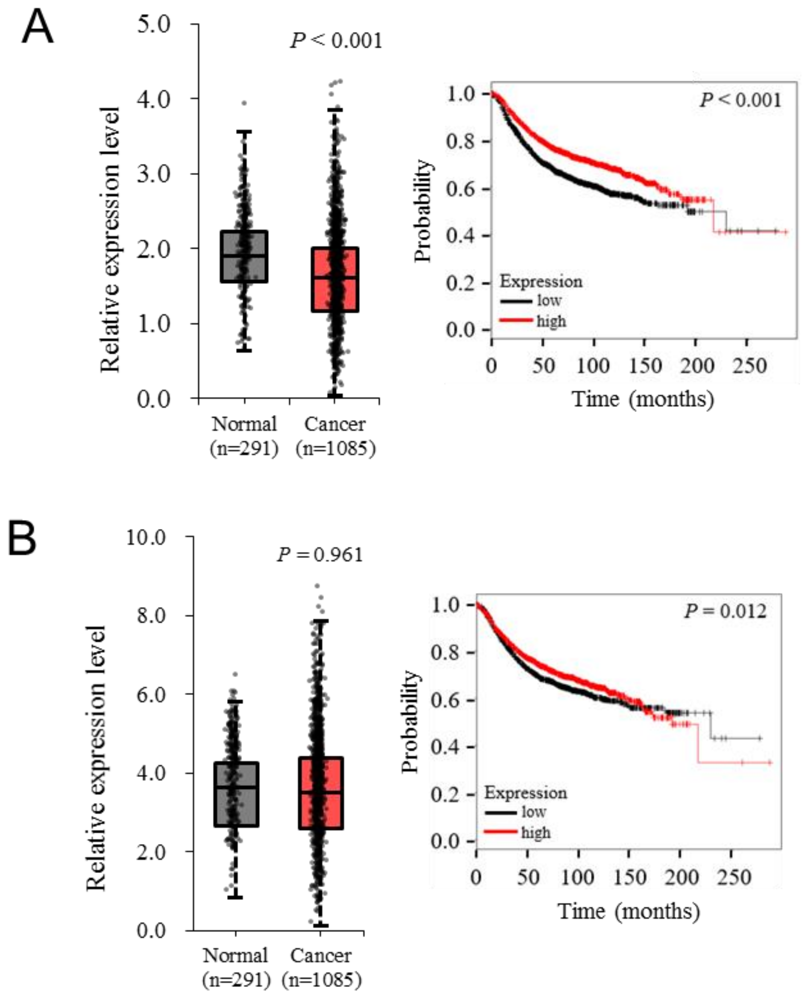

Figure S3. Kaplan–Meier survival analysis of breast cancer patients with altered expression of DAGLA and CEACAM1. (**A**) DAGLA is downregulated in breast cancer tissues compared to normal tissues, analyzed by box plot (left). Higher expression of DAGLA is significantly associated with a higher DMFS compared to lower expression (right). (**B**) For CEACAM1, no significant difference in either expression or DMSF was found between normal and cancer tissues. DMFS stands for distant metastasis free survival, meaning the period until metastasis is detected. Samples were stratified into two quantiles based on gene expression, low (gray) and high (red).

Fig. 6A

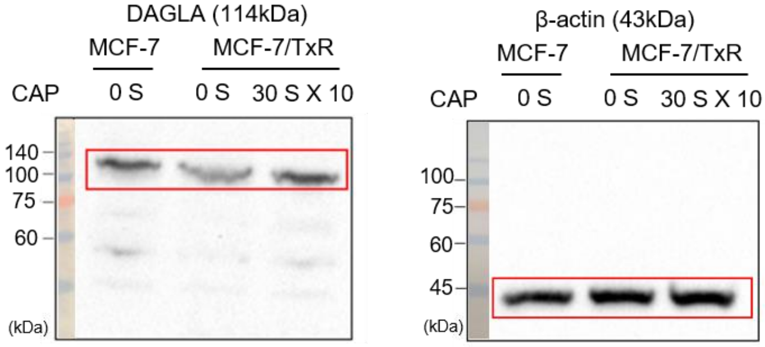

cell line	MCF-7	MCF-7/TxR	
PLASMA	0S	0S	30S x10
β-actin	11,574,376.00	13,091,020.00	13,047,160.00
DAGLA	9,318,288.00	7,707,548.00	9,054,968.00

Fig. 6B

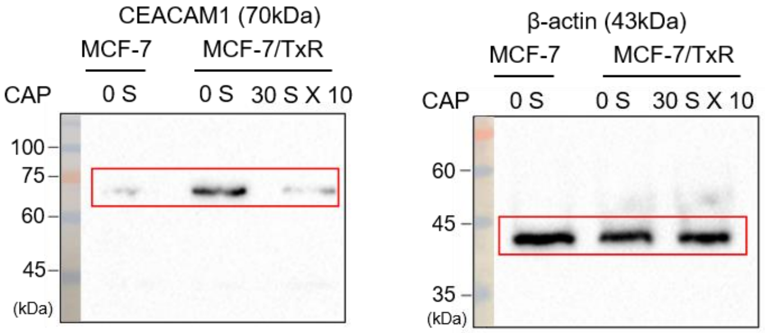

cell line	MCF-7	MCF-7/TxR	
PLASMA	0S	0S	30S x10
β-actin	6,105,432.00	3,957,516.00	4,258,412.00
CEACAM1	532,660.00	4,013,164.00	777,276.00

Figure S4. *Cont.*

Fig. 6C

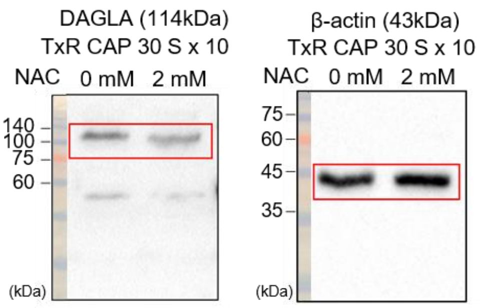

cell line	MCF-7/TxR	
NAC	0mM	2mM
β-actin	2,260,692.00	2,706,308.00
DAGLA	4,342,148.00	3,547,144.00

Fig. 6D

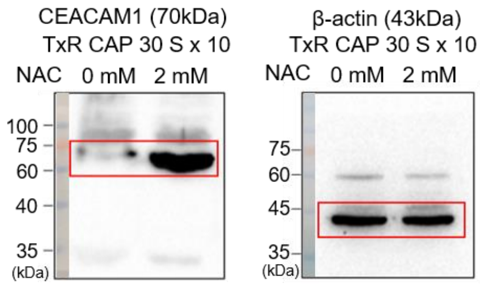

cell line	MCF-7/TxR	
NAC	0mM	2mM
β-actin	8,988,948.00	7,781,276.00
CEACAM1	14,689,992.20	310,123,318.84

Figure S4. Uncropped blots corresponding to Western blots in the main text. Boxed areas correspond to the images in the main text.
